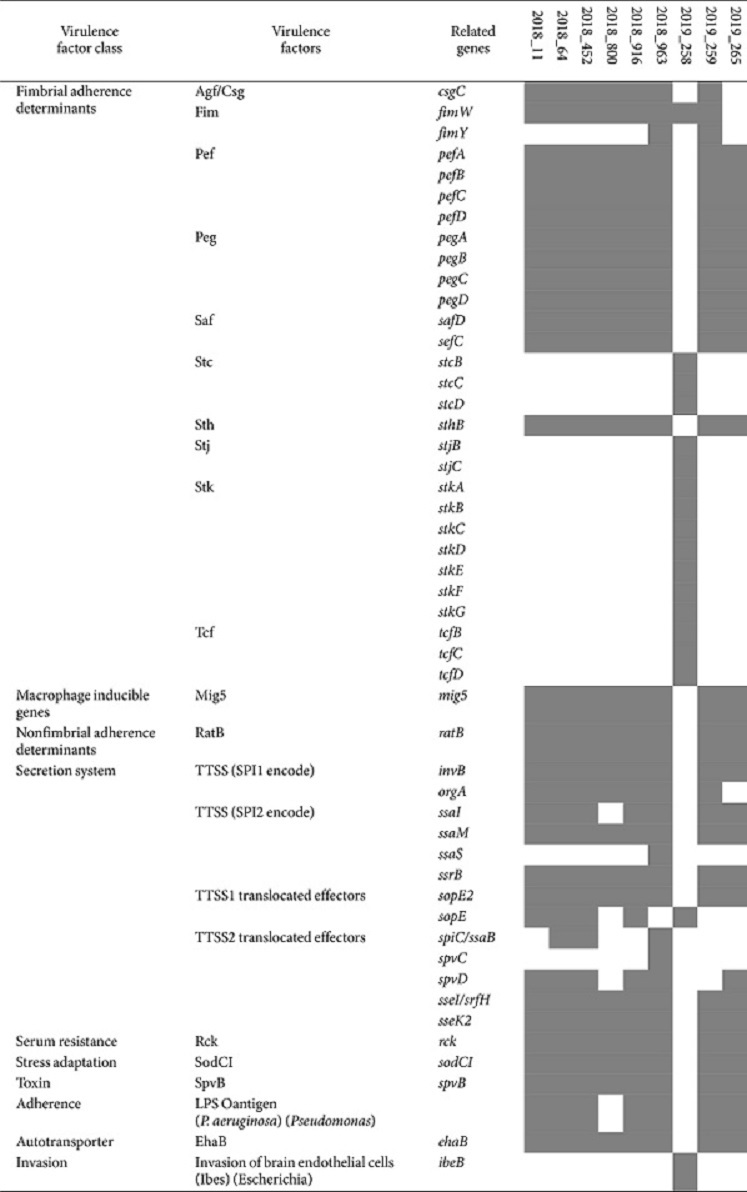# Genetic Characteristics of Extended-Spectrum Beta-Lactamase-Producing *Salmonella* Isolated from Retail Meats in South Korea

**DOI:** 10.4014/jmb.2312.12018

**Published:** 2024-03-22

**Authors:** Haiseong Kang, Hansol Kim, Hyochin Kim, Ji Hye Jeon, Seokhwan Kim, Yongchjun Park, Soon Han Kim

**Affiliations:** 1 Food Microbiology Division, Food Safety Evaluation Department, National Institute of Food and Drug Safety Evaluation, Cheongju 28159, Republic of Korea; 2 Food Standard Division, Ministry of Food and Drug Safety, Cheongju 28159, Republic of Korea

**Keywords:** Antimicrobial resistance, extended-spectrum beta-lactamase, *Salmonella* spp., genetic characteristics, whole-genome sequencing, prevalence

## Abstract

Earlier studies have validated the isolation of extended-spectrum beta-lactamase-producing *Salmonella* (ESBL-Sal) strains from food. While poultry is recognized as a reservoir for *Salmonella* contamination, pertinent data regarding ESBL-Sal remains limited. Consequently, the Ministry of Food and Drug Safety has isolated *Salmonella* spp. from retail meat and evaluated their antibiotic susceptibility and genetic characteristics via whole-genome sequencing. To further elucidate these aspects, this study investigates the prevalence, antibiotic resistance profiles, genomic characteristics, and homology of ESBL-Sal spp. obtained from livestock-derived products in South Korean retail outlets. A total of 653 *Salmonella* spp. were isolated from 1,876 meat samples, including 509 beef, 503 pork, 555 chicken, and 309 duck samples. The prevalence rates of *Salmonella* were 0.0%, 1.4%, 17.5%, and 28.2% in the beef, pork, chicken, and duck samples, respectively. ESBL-Sal was exclusively identified in poultry meat, with a prevalence of 1.4% in the chicken samples (8/555) and 0.3% in the duck samples (1/309). All ESBL-Sal strains carried the *bla*_CTX-M-1_ gene and exhibited resistance to ampicillin, ceftiofur, ceftazidime, nalidixic acid, and tetracycline. Eight ESBL-Sal isolates were identified as *S. Enteritidis* with sequence type (ST) 11. The major plasmid replicons of the Enteritidis-ST11 strains were IncFIB(S) and IncFII(S), carrying antimicrobial resistance genes (β-lactam, tetracycline, and aminoglycoside) and 166 virulence factor genes. The results of this study provide valuable insights for the surveillance and monitoring of ESBL-Sal in South Korean food chain.

## Introduction

*Salmonella* is a predominant etiological agent of diarrhea, constituting a pervasive global health concern with an annual incidence of 1.9 billion cases worldwide [1]. Moreover, its substantial contribution to diarrheal diseases surpasses that of other enteric pathogens [2]. The main sources of *Salmonella* infection include contaminated water, eggs, and meats [3]. Contaminated meat is also an important reservoir of antibiotic-resistant genes (ARGs)[4]. The dissemination of antibiotic-resistant bacteria (ARB) through the food chain poses a substantial risk as it can introduce these pathogens into the human gastrointestinal tract [5]. Therefore, meat contaminated with antibiotic-resistant *Salmonella* has emerged as a significant threat to human health worldwide.

Various antibiotics have been extensively employed for disease prevention and treatment in the livestock industry globally [6]. Notably, fluoroquinolones and cephalosporins have been prominently utilized for treating *Salmonella* infections [7]. Simultaneously, there has been a discernible increase in the application of beta-lactam antibiotics for treating *Salmonella* infections [8]. This heightened usage can exert a selective pressure conducive to the proliferation of extended-spectrum beta-lactamase (ESBL) bacteria [9]. Certain ESBL genes, located on plasmids or prophages, facilitate horizontal gene transfer to non-ESBL bacteria [4, 5]. Consequently, food contaminated with ESBL-producing *Salmonella* (ESBL-Sal) may serve as a reservoir for the spread of antimicrobial resistance (AMR) within the local community.

Previous studies have demonstrated that ESBL-Sal strains can be isolated from food samples [10-13]. Although poultry is widely acknowledged as a main source for *Salmonella* contamination, the available data on ESBL-Sal in this context remains limited [12, 14]. Therefore, to address this gap, the Ministry of Food and Drug Safety (MFDS) has isolated *Salmonella* spp. from retail meat samples, assessed their susceptibility to antibiotics, particularly beta-lactams, and comprehensively characterized their genetics through whole-genome sequencing (WGS). In pursuit of these objectives, the present study was undertaken to determine the antimicrobial prevalence, genetic profiles, and homology of ESBL-Sal in various meat types in South Korea between 2018 and 2019. The findings of this study contribute valuable insights for formulating policies to prevent the spread of antibiotic-resistant *Salmonella*.

## Materials and Methods

### Sample Collection

Between February 2018 and November 2019, a total of 1,876 meat samples comprising 509 beef, 503 pork, 555 chicken, and 309 duck were collected from retail markets in South Korea. To ensure comprehensive coverage, we divided South Korea into five zones: Seoul/Gyeonggi-do, Gyeongsang-do, Jeolla-do, Chungcheong-do, and Gangwon-do (Table 1). All samples were collected and transported under refrigerated conditions.

Isolation and Identification of *Salmonella* spp.

*Salmonella* spp. were isolated using an analytical method certified by the MFDS Food Code (https://www.mfds.go.kr/eng/brd/m_15/view.do?seq=72437). Briefly, 25 g of each sample was homogenized for approximately 30 s with 225 mL of buffered peptone water (Merck, USA) and incubated at 37°C for approximately 24 h. Subsequently, 0.1 mL of culture was inoculated into 10 mL of Rappaport–Vassiliadis broth (BD, USA), and an additional 1 mL of culture was inoculated into 10 mL of tetrathionate broth (MBcell, Republic of Korea). Both inoculated broths were subjected to a secondary incubation for 24 h at 42°C and 37°C, respectively. After incubation, the secondary culture solution was spread on XLD agar (Oxoid, UK) and brilliant green sulfa agar (Remel, UK) and incubated at 37°C for 24 h. Next, a typical colony was selected, spread on tryptone soya agar (Oxoid), and incubated at 37°C for 24 h. Vitek MS (bioMérieux, France) was used to identify the cultured bacteria following the manufacturer’s instructions.

### Antibiotic Susceptibility Test

All strains confirmed as *Salmonella* spp. were subjected to an antibiotic minimal inhibitory concentration (MIC) assay using the KRNV5F panel (TREK Diagnostic Systems, USA) following the manufacturer’s instructions. *E. coli* ATCC 25922 was used as a reference strain. The MIC assay encompassed a range of antibiotics, including amoxicillin/clavulanic acid (2/1–32/16 μg/mL), ampicillin (2–64 μg/mL), cefepime (0.25–16 μg/mL), ceftiofur (0.5–8 μg/mL), cefoxitin (1–32 μg/mL), ceftazidime (1–16 μg/mL), chloramphenicol (2–64 μg/mL), ciprofloxacin (0.12–16 μg/mL), colistin (2–16 μg/mL), gentamicin (1–64 μg/mL), meropenem (0.25–4 μg/mL), nalidixic acid (2–128 μg/mL), streptomycin (16–128 μg/mL), sulfisoxazole (16–256 μg/mL), tetracycline (2–128 μg/mL), and trimethoprim/sulfamethoxazole (0.12/2.38–4/76 μg/mL). The MICs for ceftiofur and streptomycin were interpreted in accordance with the National Antimicrobial Resistance Monitoring System breakpoints [15], whereas other antibiotics were interpreted in accordance with the Clinical and Laboratory Standards Institute breakpoints [16].

### ESBL Production Test

For cases exhibiting ceftiofur resistance and ceftazidime MIC ≥ 2 μg/mL, the ESBL phenotype was analyzed via broth microdilution using the ESB1F panel (TREK Diagnostic Systems) following the manufacturer’s instructions. The ESBL phenotype strain was characterized by an eight-fold reduction in cefotaxime and ceftazidime MICs when tested in combination with clavulanate, compared to the MICs observed in the absence of clavulanate.

### Whole Genome Sequencing and Sequence Analysis

Confirmed ESBL phenotype strains (*n* = 9) were subjected to WGS at Senigen Inc. (SRepublic of Korea). WGS was performed to determine serotypes, ARGs, plasmid replicons, multilocus sequence typing (MLST), core genome MLST (cgMLST), and virulence genes. Briefly, bacterial genomic DNA was extracted using the UltraClean Microbial DNA Isolation Kit (MoBio Laboratories Inc., USA) following the manufacturer’s instructions. Sequencing was performed on an Illumina MiSeq desktop sequencer (Illumina Inc., USA). WGS was performed using 300 bp paired-end sequencing. Raw reads were assembled using the SPAdes genome assembler version 3.13.0. Contigs less than 200 bp length and 5× sequencing depth were removed. Assembled contigs maintained an average sequencing depth of 130×.

### Nucleotide Sequence Accession Numbers

The raw WGS data have been deposited in GenBank under the BioProject PRJNA1002882, with the following biosample accession numbers: SAMN36866225 (2018-11), SAMN36866226 (2018-64), SAMN36866227 (2018-452), SAMN36866228 (2018-800), SAMN36866229 (2018-916), SAMN36866230 (2018-963), SAMN36866231 (2019-258), SAMN36866232 (2019-259), and SAMN36866233 (2019-265).

### Phylogenetic Analysis

Homology among ESBL phenotype strains was compared using MLST and cgMLST. The MLST database (http://pubmlst.org/database/) referenced seven housekeeping genes (*aroC*, *dnaN*, *hemD*, *hisD*, *purE*, *sucA*, and *thrA*), and MLST 2.0 assigned sequence types to strains. For cgMLST analysis, raw sequence data files were uploaded to cgMLSTfinder 1.2 at the Center for Genomic Epidemiology [17], and allelic profiles were predicted. A minimum spanning tree based on the allelic profile of cgMLST was constructed using GrapeTree version 1.5.0.

### In Silico Characterization of WGS

ARGs were predicted using ResFinder 4.1, with 90% as the minimum for identity and 60% as the cut-off query length. Plasmid typing was predicted using Plasmidfinder 2.1, with 95% as the minimum for identity and 60% as the cut-off query length. *Salmonella* pathogenicity islands were predicted using SPIFinder 2.0, with 95% as the minimum for identity and 60% as the minimum for coverage. *Salmonella* serotypes were predicted using SeqSero 1.2. Virulence factors were identified using the Virulence Factor Database [18], with 90% as the minimum for identity and 50% as the minimum for coverage.

### Statistical Analysis

Chi-square tests on the Epitools website were employed to assess the significance of proportional differences within the 95% confidence interval scale [19]. Briefly, we compared *Salmonella* isolates from chicken and duck samples, specifically examining antibiotic-resistant and antibiotic-sensitive strains.

## Results

### Prevalence of ESBL-Producing *Salmonella* in Meat Samples

A total of 191 *Salmonella* strains were isolated from 1,864 meat samples. The prevalence rates of *Salmonella* were 28.2%, 17.5%, and 1.4% in the duck, chicken, and pork samples, respectively. No *Salmonella* strain was isolated from beef. Furthermore, the prevalence of ESBL-Sal was 1.4% in the chicken samples and 0.3% in the duck samples (Table 2). Only nine strains were identified as ESBL-producing *Salmonella*. Thus, poultry meat exhibited a higher prevalence of *Salmonella* and ESBL-Sal.

### Antimicrobial Resistance Patterns of *Salmonella*

A chi-square test (*p* < 0.05) revealed significant differences in AMR between chicken and duck isolates for ten antibiotics, namely, amoxicillin/clavulanic acid, ampicillin, cefepime, ceftiofur, ceftazidime, nalidixic acid, streptomycin, sulfisoxazole, tetracycline, and trimethoprim/sulfamethoxazole (Table 3). Among poultry samples, the most prevalent resistance to non-beta lactam antibiotics was observed with nalidixic acid and tetracycline. *Salmonella* strains isolated from pork were resistant to only four antibiotics: ampicillin, streptomycin, sulfisoxazole, and tetracycline.

### Serotyping and Antimicrobial Resistance of ESBL-Producing *Salmonella*

The serotypes of the nine isolates were investigated using SeqSero1.2. Among them, eight strains (2018_11, 2018_64, 2018_452, 2018_800, 2018_916, 2018_963, 2019_259, and 2019_265) were predicted to be *S. Enteritidis* (Table 4). Notably, all *S. Enteritidis* strains exhibited resistance to ampicillin, ceftiofur, ceftazidime, nalidixic acid, and tetracycline. Furthermore, *S. Enteritidis* demonstrated a resistance rate of 87.5% to cefepime and gentamicin. The serotype of the 2019_258 strain could not be predicted. This strain was predicted based solely on O-antigen 7, resulting in an antigenic profile of 7:-:-. The 2019_258 strain was resistant to ampicillin, ceftiofur, ceftazidime, nalidixic acid, streptomycin, sulfisoxazole, and tetracycline.

### Phylogenetic Analysis

**Fig. 1:**
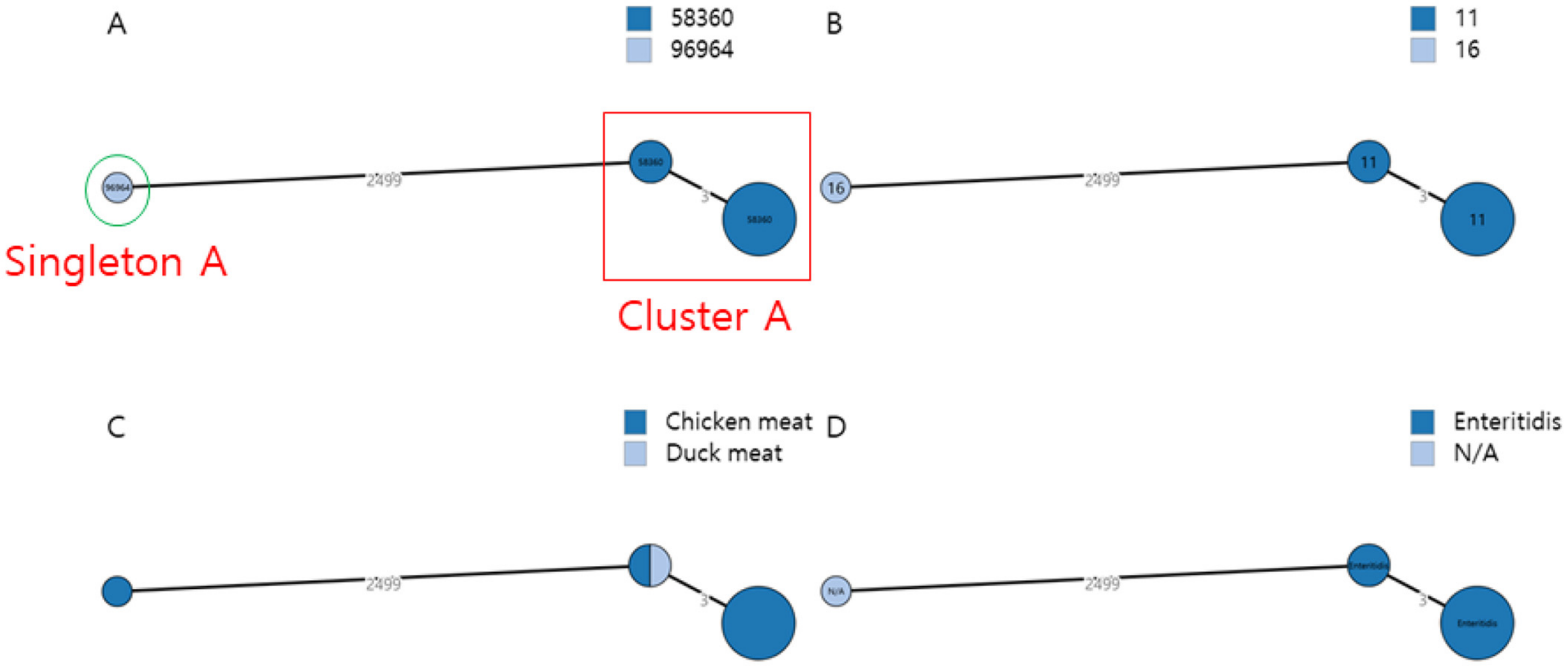
The minimum spanning tree was based core genome multilocus sequence typing. Nodes colour were divided; each (**A**) cgMLST (**B**) MLST (**C**) source (**D**) serotype.

The homology among the nine ESBL-Sal isolates was assessed using cgMLSTFinder (version 1.2) and GrapeTree (version 1.5.0) (Fig. 1). In the minimum spanning tree, these nine ESBL-Sal isolates were divided into one cluster and one singleton. The singleton, identified as the strain 2019_258 chicken isolate, was assigned cgST96964 and ST16. The cluster comprised eight strains (2018_11, 2018_64, 2018_452, 2018_800, 2018_916, 2018_963, 2019_259, and 2019_265), all predicted to be cgST58360, ST11, and *S. Enteritidis*. These cluster strains were isolated from seven chickens and one duck. Notably, an average of 2,499 allelic differences were observed between the cluster and singleton.

### Detection of Antimicrobial Resistance and Plasmid Genes

We identified five distinct plasmid replicon types, namely, IncFIB(S) (reference accession no: FN432031), IncFII(S) (CP000858), IncQ1 (M28829), IncHI2 (BX664015), and IncHI2A (BX664015) (Table 5). All *S. Enteritidis* strains carried IncFIB(S) and IncFII(S). Notably, the 2018_916 strain carried IncQ1 in addition to IncFIB(S) and IncFII(S). The 2019_258 strain carried three plasmid replicons, namely, IncQ1, IncHI2, and IncHI2A. Meanwhile, IncHI2 and IncHI2A were observed exclusively in the nine ESBL-Sal strains. Our findings revealed the presence of beta-lactam, tetracycline, aminoglycoside (five distinct types), and sulfonamide genes (Table 5). All ESBL-Sal carried *bla*_CTX-M-15_, *tet*(A), and *aac(6')-Iaa*. Among the cluster strains, seven (excluding the 2018_963 strain) carried *acc(3)-lld*. Furthermore, the 2018_916 and 2019_258 strains carried five (*aac(3)-IId*, *aph(3')-Ia*, *aph(3’)-Ib*, *aph(6)-Id*, and *sul2*) and three (*aph(3'')-Ib*, *aph(6)-Id*, and *sul2*) ARGs, respectively.

### Detection of Pathogenicity Islands and Virulence Factors

All ESBL-Sal strains carried identical pathogenicity island genes. The genes detected included SPI1, SPI2, SPI3, SPI4, SPI5, SPI9, SPI10, SPI13, SPI14, C63PI, and CS54. A comprehensive assessment of virulence factors revealed 182 pertinent genes in the nine ESBL-Sal stains. Among these, 133 were consistently detected (Table 6), whereas 49 exhibited different profiles (Table 7). The identified virulence factor classes included fimbrial adherence determinants, macrophage-inducible genes, magnesium uptake, non-fimbrial adherence determinants, regulation, secretion system, serum resistance, stress adaptation, toxin, adherence, autotransporter, and invasion. Among the nine strains, eight (cgST58360) exhibited comparable virulence factor profiles. This subset of eight strains (cgST58360) was investigated, focusing on fimbrial adherence determinants (*pef*, *peg*, *saf*, and *sth*), macrophage-inducible genes (*mig5*), nonfimbrial adherence determinants (*ratB*), secretion system (TTSS (encoded by SPI1), TTSS (encoded by SPI2), translocated effectors of TTSS1 and TTSS2), serum resistance (*rck*), stress adaptation (*sodCI*), toxin (*spvB*), and autotransporter (*ehaB*).

## Discussion

In this study, we isolated 97 and 87 *Salmonella* strains from 555 chicken and 309 duck samples, respectively. The prevalence of *Salmonella* was 17.5% in the chicken samples and 28.2% in the duck samples. The chicken isolates exhibited resistance rates of 22.7% and 21.6% to ceftiofur and ceftazidime, respectively. However, the duck isolates of *Salmonella* exhibited resistance rates of 2.3% and 2.3% to ceftiofur and ceftazidime, respectively. The prevalence of ESBL-Sal was 1.4% in the chicken samples and 0.3% in the duck samples, respectively. Samples were collected between 2018 and 2019, with a roughly equal distribution of samples collected repeatedly. However, substantial disparities were observed in the prevalence rate of *Salmonella*, ESBL-Sal, and 3rd-generation cephalosporin AMR between the chicken and duck meat samples. The introduction of antibiotics decreased microbial diversity; high antibiotic selective pressures increased the abundance of ARGs but coincided with a reduction in the diversity of ARGs [20]. These findings suggest a higher likelihood of *Salmonella* contamination with a high AMR level in chicken meat than in duck meat, indicating a potentially greater use of antibiotics in chicken production.

Among the nine ESBL-Sal strains, eight were identified as Enteritidis-ST11 and one as ST16, albeit with an indeterminate serotype prediction for ST16. Among the Enteritidis-ST11 strains, seven were isolated from the chicken samples and one from the duck samples. The ST16 strain was isolated exclusively from chicken meat. ST11 and ST16 were the most common *Salmonella* strains isolated from chicken meat in South Korea, consistent with previous studies [21, 22]. Moreover, the cgMLST assay revealed that the Enteritidis-ST11 and ST16 strains consisted of one cluster and one singleton, respectively. MLST and cgMLST showed similar homologies. All ESBL-Sal isolates were resistant to ampicillin, ceftiofur, ceftazidime, nalidixic acid, and tetracycline and carried *bla*_CTX-M-15_ belonging to the *bla*_CTX-M-1_ group. The Enteritidis-ST11 group exhibited an 87.5% (7/8) resistance rate to cefepime and gentamicin. Enteritidis carrying the *bla*_CTX-M-1_ gene exhibits resistance to ampicillin, ceftiofur, ceftazidime, nalidixic acid, tetracycline, and gentamicin [21, 23], consistent with our findings. Eight ARGs, including *bla*_CTX-M-15_, were detected in this study. Moreover, *S. Enteritidis* strains isolated from chickens harbored comparable ARGs. Consistent with the results outlined earlier, the detection rate of *bla*_CTX-M-15_, *tet*(A), and *aac(6')-laa* was 100% (8/8), and that of *acc(3)-lld* was 87.5% (7/8). However, *S. Enteritidis* strains isolated from duck samples harbored four additional ARGs: *aph(3')-la*, *aph(3'')-lb*, *aph(6)-ld*, and *sul2*. The isolated chicken-derived ST16 strain harbored *bla*_CTX-M-15_, *tet*(A), *aac(6')-laa*, *aph(3'')-lb*, *aph(6)-ld*, and *sul2*. All ESBL-Sal strains exhibited 11 identical pathogenicity islands, encompassing SPI1, SPI2, SPI3, SPI4, SPI5, SPI9, SPI10, SPI13, SPI14, C63PI, and CS54. SPI-1 is associated with target cell invasion during the infection initiation stage, whereas SPI-2 affects a broad range of systemic infection functions [24]. *S. Enteritidis* strains contained 166 genes among the 182 virulence factor genes, 94% (156/166) of which were similar. The 2019_258 strain contained 151 of the virulence factor genes, of which 16 genes were detected exclusively.

In this study, *S. Enteritidis* was considered a representative serotype from poultry, which may cause ESBL-Sal infections in humans. The ESBL-Sal strain isolated from poultry meat in most parts of the country exhibited a specific sequence type and serotype of Enteritidis-ST11. This predominant group displayed one cluster in the cgMLST analysis. Moreover, these strains exhibited resistance to ampicillin, ceftiofur, ceftazidime, nalidixic acid, and tetracycline. Genomic characterization revealed the presence of beta-lactam, tetracycline, and aminoglycoside resistance genes in two plasmid replicons, namely, IncFIB(S) and IncFII(S). WGS analysis revealed 11 identical pathogenicity islands and 156 identical virulence factor genes.

This study has certain limitations. The study design was limited in the context of the sampling of ESBL-Sal isolates. Utilizing a *Salmonella* selective culture medium, we isolated a maximum of one *Salmonella* spp. per meat sample. Consequently, this study may differ from studies that used an ESBL-selective culture medium to isolate ESBL bacteria. Moreover, most *Salmonella* serotypes could not be determined.

Despite these limitations, this study provides valuable insights into the WGS characteristics of ESBL-Sal in South Korean retail meat. The use of MLST and cgMLST allowed for homology determination, which could enable traceback investigations in ESBL-Sal poisoning. Additionally, we identified various major genes, including those related to AMR, plasmid replicon, identical pathogenicity island, and virulence factor genes. Ultimately, this research offers valuable information that can aid in preventing the continued spread of ESBL-Sal.

In summary, we investigated the prevalence and genetic characteristics of ESBL-Sal strains isolated from retail meat in South Korea between 2018 and 2019. The findings suggest that poultry might be the main reservoir for ESBL-Sal, extending beyond *Salmonella* spp. The predominant ESBL-Sal type identified was Enteritidis-ST11, possessing two plasmid replicons and various antibiotic and virulence factor genes. This finding is significant given previous reports of an outbreak in South Korea involving IncFII plasmids carrying the *bla*_CTX-M-15_ gene in Enteritidis-ST11 isolated from humans [21]. Thus, retail chicken meat may serve as a potential contagion channel for ESBL-Sal poisoning. Accordingly, continuous monitoring of the poultry industry is essential to prevent the spread of ESBL-Sal. Moreover, further surveillance of ARBs in the supply chain of livestock products sold at retail outlets is warranted.

## Figures and Tables

**Table 1:** Sample tested in this study.

Meat type	Region					
	Seoul and Gyeonggi-do	Gyeongsang-do	Chungcheong-do	Jeolla-do	Gangwon-do	Total
Beef	91	137	117	101	63	509
Pork	90	134	124	103	52	503
Chicken	97	194	131	73	60	555
Duck	39	132	61	56	21	309
Total	317	597	433	333	196	1876

**Table 2:** Prevalence of *Salmonella* spp. and ESBL-producing *Salmonella* spp. among retail meats in Korea, 2018-2019.

% (number of positive samples/number of tested samples)		
Meat type	*Salmonella* spp.	ESBL-producing *Salmonella* spp.
Beef	0.0 (00/509)	0.0 (0/509)
Pork	1.4 (7/503)	0.0 (0/503)
Chicken	17.5 (97/555)	1.4 (8/555)
Duck	28.2 (87/309)	0.3 (1/309)
Total	10.2 (191/1876)	0.5 (9/1876)

**Table 3:** Antibiotic resistance profiles of *Salmonella* spp. isolated from meat samples.

Antibiotic	% (number of resistant strains)				
	Pork (*n* = 7)	Chicken meat (*n* = 97)	Duck meat (*n* = 87)	Subtotal (*n* = 191)	*P*-value
Amoxicillin/clavulanic acid	0.0(0)	9.3(9)	1.1(1)	5.2(10)	0.0355
Ampicillin	14.3(1)	59.8(58)	19.5(17)	39.8(76)	<0.0001
Cefepime	0.0(0)	11.3(11)	1.1(1)	6.3(12)	0.0126
Ceftiofur	0.0(0)	22.7(22)	2.3(2)	12.6(24)	0.0001
Cefoxitin	0.0(0)	5.2(5)	1.1(1)	3.1(6)	0.2663
Ceftazidime	0.0(0)	21.6(21)	2.3(2)	12.0(23)	0.0002
Chloramphenicol	0.0(0)	12.4(12)	4.6(4)	8.4(16)	0.1082
Ciprofloxacin	0.0(0)	1.0(1)	3.4(3)	2.1(4)	0.5377
Colistin	0.0(0)	16.5(16)	12.6(11)	14.1(27)	0.5972
Gentamicin	0.0(0)	10.3(10)	2.3(2)	6.3(12)	0.0577
Meropenem	0.0(0)	0.0(0)	0.0(0)	0.0(0)	N/A
Nalidixic acid	0.0(0)	88.7(86)	39.1(34)	62.8(120)	<0.0001
Streptomycin	28.6(2)	36.1(35)	14.9(13)	26.2(50)	0.002
Sulfisoxazole	14.3(1)	43.3(42)	14.9(13)	29.3(56)	<0.0001
Tetracycline	28.6(2)	40.2(39)	17.2(15)	29.3(56)	0.0011
Trimethoprim/Sulfamethoxazole	0.0(0)	13.4(13)	3.4(3)	8.4(16)	0.0331

**Table 4:** Antimicrobial resistance profiles of ESBL-producing *Salmonella* spp.

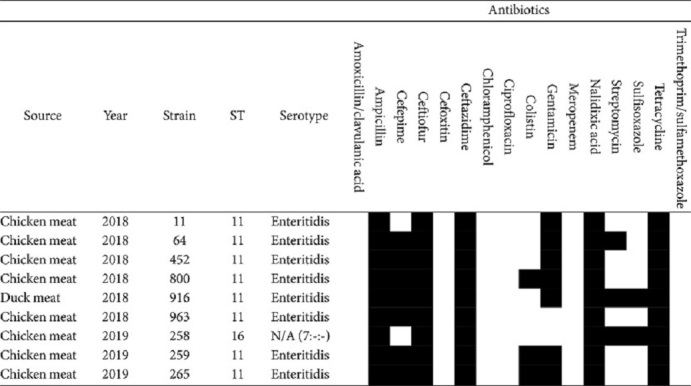

**Table 5:** Identified antibiotic resistance and plasmid genes profile of ESBL-producing *Salmonella* spp.

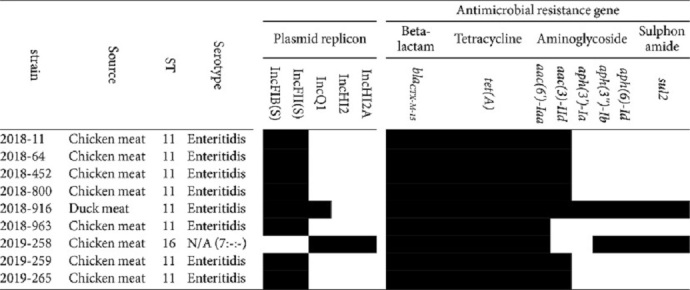

**Table 6:** Identified common virulence factor genes of ESBL-producing *Salmonella* spp.

VF class	Virulence factor	Related gene
Fimbrial adherence determinants	Agf/Csg	*csgA, csgB, csgD, csgE, csgF, csgG*
	Bcf	*bcfA, bcfB, bcfC, bcfD, bcfE, bcfF, bcfG*
	Fim	*fimA, fimC, fimD, fimF, fimH, fimI, fimZ*
	Lpf	*lpfA, lpfB, lpfC, lpfD, lpfE*
	Saf	*safB*
	Saf	*safC*
	Stb	*stbA, stbB, stbC, stbD, stbE*
	Std	*stdA, stdB, stdC*
	Ste	*steA, steB, steC, steD, steE, steF*
	Stf	*stfA, stfC, stfD, stfE, stfF, stfG*
	Sth	*sthA, sthC, sthD, sthE*
	Sti	*stiA, stiB, stiC, stiH*
Macrophage inducible genes	Mig14	*mig14*
Magnesium uptake	Mg2+ transport	*mgtB, mgtC*
Nonfimbrial adherence determinants	MisL	*misL*
	ShdA	*shdA*
	SinH	*sinH*
Regulation	PhoPQ	*phoP, phoQ*
Secretion system	TTSS (SPI1 encode)	*hilA, hilC, hilD, iacP, iagB, invA, invC, invE, invF, invG, invH, invI, invJ, orgB, orgC, prgH, prgI, prgJ, prgK, sicA, sicP, sipD, spaO, spaP, spaQ, spaR, spaS, sprB*
	TTSS (SPI2 encode)	*ssaC, ssaD, ssaE, ssaG, ssaH, ssaJ, ssaK, ssaL, ssaN, ssaO, ssaP, ssaQ, ssaR, ssaT, ssaU, ssaV, sscA, sscB, sseB, sseC, sseD, sseE, ssrA*
	TTSS effectors translocated via both systems	*slrP*
	TTSS1 translocated effectors	*avrA, sipA, sipB, sipC, sopA, sopB/sigD, sopD, sptP*
	TTSS2 translocated effectors	*pipB2, pipB, sifA, sifB, sseF, sseG, sseJ, sseK1, sseL, sspH2*

**Table 7:** Identified virulence factor genes of ESBL-producing *Salmonella* spp. except common virulence factor genes.